# BTV Remodels the oISG15-Association Proteome

**DOI:** 10.3390/microorganisms14081855

**Published:** 2026-08-20

**Authors:** Di Kang, Qinxue Gao, Mingxin Zhang, Rui Ha, Xinbing Hu, Meng Li, Zhongming Chen, Shijun Bao

**Affiliations:** 1 College of Veterinary Medicine, Gansu Agricultural University, Lanzhou 730070, China; 15730939123@163.com (Q.G.); mingxinzhang123@163.com (M.Z.); harui0419@163.com (R.H.); limeng_lm5@163.com (M.L.); 15597173006@163.com (Z.C.); bsjdy@126.com (S.B.); 2 Laboratory Animal Center, Air Force Medical University, Xi’an 710032, China; 3 State Key Laboratory of Animal Disease Control and Prevention, College of Veterinary Medicine, Lanzhou University, Lanzhou Veterinary Research Institute, Chinese Academy of Agricultural Sciences, Lanzhou 730046, China; xinbinghu123@163.com

**Keywords:** ISG15, bluetongue virus, proteomics, autophagy, mitochondrial metabolism

## Abstract

ISG15 is an interferon-induced ubiquitin-like protein that exerts diverse functions during viral infection. We previously reported that ovine ISG15 (oISG15) promoted bluetongue virus (BTV) replication by stabilizing viral NS1 and VP4 in an ISGylation-independent manner, although the mechanism remained unknown. Here, using the generated anti-oISG15 antibody, we performed immunoprecipitation (IP) coupled with label-free Liquid Chromatography-Tandem Mass Spectrometry (LC–MS/MS) to characterize the oISG15-associated proteome in BTV-infected and uninfected cells. The results showed that BTV infection markedly remodeled the oISG15 association landscape, with decreased enrichment of several autophagy- and trafficking-related proteins and increased enrichment of mitochondrial metabolic proteins in oISG15 immunoprecipitates. Functionally, oISG15 overexpression induced modest alterations in autophagy-related markers in uninfected cells yet attenuated these markers’ abundance during BTV infection. Together, this study revealed BTV-induced changes in the oISG15-associated proteome that coincided with alterations in autophagy-related markers.

## 1. Introduction

Bluetongue virus (BTV) is a representative member of the genus *Orbivirus* in the family *Reoviridae*. It is an economically important pathogen in ruminants transmitted by *Culicoides* mosquitoes [1]. The viral particle is composed of an outer capsid (VP2 and VP5), an inner capsid (VP3 and VP7), and a transcription complex including VP1, VP4, and VP6. In addition to structural proteins, BTV genome encodes five non-structural proteins (NS1, NS2, NS3/3A, NS4, and NS5). NS1 is the most abundantly expressed viral protein and the predominant cytoplasmic protein prior to the completion of virus replication [2]. It is a highly conserved tubule-forming protein and acts as the positive regulator of viral mRNA translation [3]. The type I interferon (IFN-I) system constitutes the first line of defense against virus infection. Among the hundreds of interferon-stimulated genes (ISGs) induced upon IFN-I signaling, ISG15 is one of the most significantly up-regulated proteins in sheep testicular cells following BTV infection, suggesting that it may be involved in the regulation of host cellular activities and BTV replication [4]. ISG15 is an ubiquitin-like protein that is covalently conjugated to the target proteins (ISGylation). In ISGylation, the ISG15 precursor is cleaved to generate mature ISG15 with a conserved C-terminal LRLRGG motif. Sequential reactions mediated by the E1 enzyme UBA7, the E2 enzyme UBE2L6, and multiple E3 ligases drive the covalent conjugation of ISG15 to substrates, whereas the protease USP18 can reverse this modification [5]. Apart from its covalent conjugation, free ISG15 also functions intracellularly and extracellularly through non-covalent protein-protein interactions [6].

The biological functions of ISG15 extend well beyond its canonical roles in regulating cellular immune response. Previous mass spectrometry (MS) analysis for ISG15 revealed that its target proteins function in diverse cellular pathways, including RNA processing, cytoskeleton organization and regulation, stress responses, and translation [7]. Takeuchi et al. overexpressed His-FLAG-tagged ISG15 and E1/E2 enzymes in HeLa cells, followed by affinity pulldown and MS identification to identify the ISGylated proteins [8]. Wong and colleagues generated FLAG-tagged ISG15-expressing A549 cells, which were used to pull down ISGylated proteins from IFN-I-stimulated cells, and identified 168 ISGylation target proteins [9]. However, immunoprecipitation (IP)-based identification of endogenous ISG15 substrates still has considerable limitations. The current ISG15 antibodies cannot selectively recognize ISGylation-modified residues, resulting in low specificity enrichment efficiency for covalently modified ISG15 substrates. To overcome the disadvantages of low abundance of ISG15-modified substrates, transient or unstable binding, and difficulty in capture, researchers have employed various methods to improve the efficiency of enriching ISGylated substrates [10,11].

Our prior study identified that oISG15 was a proviral host factor that relies on an ISGylation-independent mechanism for BTV, given that mutating conserved conjugation sites failed to eliminate its promoting effect. We found that free oISG15 interacts with VP4 and NS1 to maintain the stability of these proteins. Furthermore, NS1 was found to undergo K63-linked ubiquitination (K63-Ub), a canonical signal for autophagic cargo recognition, leading us to hypothesize that oISG15 monomers might influence the susceptibility of viral proteins to autophagic degradation [12]. Previous reports revealed that free or conjugated ISG15 can interact with SQSTM1/p62, potentially promoting autophagy of ISG15-conjugates [13]. These studies depicted the diverse roles of ISG15 in protein degradation across distinct viral infections, yet the global association landscape of oISG15 during BTV infection remained unexplored.

To address this, we generated the anti-oISG15 antibody, performed IP coupled with label-free Liquid Chromatography-Tandem Mass Spectrometry (LC–MS/MS), and compared the oISG15-associated proteins in the presence and absence of BTV infection. We found that BTV infection was accompanied by lower oISG15 association with several autophagy- and trafficking-related components and higher association with mitochondrial metabolic proteins, including PDC subunits and VDAC1. We further examined whether the remodeling of oISG15 association was accompanied by attenuation of the BTV-induced autophagic response, changes in mitochondrial quality-control markers, and viral protein accumulation. Together, these analyses provide a proteome-wide view of BTV-induced remodeling of the oISG15 association network and its potential relationship with autophagy and mitochondrial quality control markers during infection.

## 2. Materials and Methods

### 2.1. Cells, Viruses, and Plasmids

The ovine-derived kidney cells (MDOK, ATCC-CRL-1633) were maintained in Dulbecco’s modified Eagle’s medium (Gibco, Grand Island, NY, USA) containing 10% FBS (Gibco, Grand Island, NY, USA). Baby hamster kidney 21 cells (BHK-21, ATCC-CCL-10) were cultured in modified Eagle’s medium (Hyclone, Logan, UT, USA) supplemented with 5% FBS. The BTV-1 strain (GS/11) was propagated in BHK-21 cells.

The codon-optimized coding sequence of oISG15 was synthesized (Sangon Biotech, Shanghai, China) and subcloned into pET-28a (+) vector with an N-terminal His tag to construct the recombinant oISG15 prokaryotic expression plasmid. The construction was confirmed by DNA sequencing, and the plasmid was named pET-oISG15.

### 2.2. Recombinant oISG15 Purification and Anti-oISG15 Antibody Generation

The pET-oISG15 was transformed into *E. coli* BL21(DE3) competent cells. A single colony was picked from LB solid plates and inoculated into liquid LB medium, followed by shaking incubation until the OD_600_ value reached 0.6–0.8. Protein expression was induced by adding isopropyl β-D-1-thiogalactopyranoside (IPTG, 1.0 mM). After induction, bacterial cells were harvested, and the cell pellets were washed and resuspended in lysis buffer (Beyotime, Shanghai, China, Cat. No. P0013Q) on ice. The lysate supernatant and precipitate were collected separately for assessment of recombinant protein solubility by SDS-PAGE.

The Ni-NTA column was equilibrated with binding buffer, after which the supernatant was loaded. The column was then washed with wash buffer. The His-tagged oISG15 protein was eluted with the elution buffer. Eluted fractions were analyzed by SDS-PAGE, and fractions containing the target protein were pooled and dialyzed against phosphate-buffered saline (PBS, pH 7.4). Protein concentration was determined by the BCA method (Thermo Fisher, Waltham, MA, USA, Cat. No. A65453).

The immunization of rabbits with recombinant oISG15 protein and the subsequent antibody purification were commissioned to Abmart Company (Shanghai, China). Briefly, after four immunization injections, serological testing verified that the titer was up to 1:12,800. Rabbits were sacrificed for cardiac blood collection, serum was separated, and specific antibodies were purified from the obtained serum.

### 2.3. ELISA, Immunofluorescence, and Immunoprecipitation–Western Blot Validation

The reactivity of the anti-oISG15 antibody was determined by indirect enzyme-linked immunosorbent assay (ELISA) and immunofluorescence assay (IFA). For the ELISA assay, the purified recombinant oISG15 protein was coated, and the coating amount was gradually diluted by multiples. After blocking with 1% bovine serum albumin (BSA), the anti-oISG15 antibody was diluted at a ratio of 1:1000 and added to each well, and the pre-immune serum was used as the negative control. For the IFA assay, MDOK cells were grown on coverslips and infected with BTV-1 at an MOI of 1; mock-infected cells served as controls. After incubation for 24 h, the samples were permeabilized with 0.5% Triton X-100 and blocked with 3% BSA. The anti-oISG15 antibody was used as primary antibody, incubating overnight at 4 °C (1:500 dilution), and then the Alexa Fluor-conjugated goat anti-rabbit (Abcam, Waltham, MA, USA, 1:1000 dilution) was incubated for 1 h at RT. The samples were incubated with DAPI (Thermo Fisher, Waltham, MA, USA, Cat. No. 62248) for 10 min after the washing steps. Images were obtained using a confocal microscope (Leica TCS SP8, Leica Microsystem, Wetzlar, Germany).

To evaluate whether the anti-oISG15 antibody could immunoprecipitate endogenous oISG15, the MDOK cells were mock-infected or infected with BTV-1 at an MOI of 1. After 24 h, the cells were lysed in NP-40 lysis buffer (Beyotime, Shanghai, China, Cat. No. P0013F). For each IP reaction, 1 mg of total protein was incubated with 4 μg of anti-oISG15 antibody at 4 °C overnight with rotation. Pre-immune rabbit serum was used in parallel as a negative IP control to assess nonspecific binding. Subsequently, 30 μL of Protein A/G magnetic beads (MedChemExpress, Monmouth Junction, NJ, USA, Cat. No. HY-K0202) were added and incubated for another 4 h at 4 °C. The beads were collected and washed five times with lysis buffer. Corresponding input lysates were analyzed in parallel to verify oISG15 abundance. The immunoprecipitates were analyzed by Western blot using anti-oISG15 antibody (1:5000 dilution).

### 2.4. Sample Preparation for LC–MS/MS Analysis

To identify oISG15-associated proteins, the MDOK cells were seeded in six 10 cm culture dishes and cultured until 80–90% confluence. Three of them were infected with BTV-1 at MOI = 1, and others served as negative controls. After incubation for 24 h, the MDOK cells were washed twice with pre-cooled PBS and lysed in IP lysis buffer as described above. The cell lysate was centrifuged at 12,000 rpm for 20 min at 4 °C to remove cell debris and insoluble precipitates, and then the supernatant was carefully transferred to a new pre-cooled centrifuge tube. After quantifying the total protein concentration of each lysate, equal amounts of protein from each sample were pre-cleared by immunoprecipitation with 5 μL of pre-immune IgG, then immunoprecipitated with 4 μg anti-oISG15 antibody using the same protocol as mentioned above. After washing five times, the beads were resuspended in 50 μL of PBS and immediately transported on dry ice for 4D label-free LC–MS/MS detection. Three independent biological replicates were included for each group. The samples of the BTV infected group were named as BTV-oISG15, and the negative control group was named as NC-oISG15.

### 2.5. 4D Label-Free LC–MS/MS Data Acquisition and Protein Quantification

The 4D label-free quantitative proteomic analysis was performed by Applied Protein Technology (Shanghai, China). Briefly, the immunoprecipitated proteins were lysed in SDT buffer (100 mM Tris-HCl, pH 7.6, 4% SDS), and then the BCA method was used for protein quantification. An amount of 20 μg of protein from each sample was subjected to trypsin digestion using filter-aided sample preparation (FASP). The resulting peptides were desalted on C18 cartridges, lyophilized, and reconstituted in 0.1% formic acid. Peptide separation was carried out on a nanoElute HPLC system (Evosep One, Evosep Biosystm, Odense, Demark) coupled to a timsTOF Pro mass spectrometer. Data were acquired in positive-ion mode using the parallel accumulation-serial fragmentation (PASEF) scheme on a timsTOF Pro mass spectrometer.

Raw MS data were processed using MaxQuant (version 1.6.14), and protein abundance was quantified using the MaxLFQ algorithm. The resulting LFQ intensities were used for downstream quantitative analysis. Missing values were not imputed. Statistical significance was assessed using a two-sided Student’s *t*-test on LFQ intensity values, followed by Benjamini-Hochberg correction for multiple testing. The statistical workflow followed the output of MaxQuant LFQ quantification provided by the proteomics service provider.

### 2.6. Bioinformatic Analysis

The LFQ intensities obtained from MaxQuant were used for statistical analysis. To systematically characterize the quantitative landscape of oISG15-association dynamics, we performed differential abundance analysis on proteins consistently detected (present in three biological replicates) in both the BTV-oISG15 and NC-oISG15 groups using a two-sided Student’s *t*-test on the LFQ intensity values. *p*-values were adjusted using the Benjamini-Hochberg procedure to control the false discovery rate. The proteins (BTV-oISG15 versus NC-oISG15) satisfying the thresholds of FDR < 0.05 and fold change (FC) ≥ 1.2 or ≤0.833 were defined as significantly differentially enriched proteins. 

However, the IP–MS data often contain biologically relevant proteins that are exclusively detected in one group (present in ≥2 out of 3 replicates in one group but absent in the other), which were classified as qualitative group-specific proteins. For bioinformatic analyses that did not require FC values, these proteins combined with the significantly differentially enriched proteins were collectively defined as differential oISG15-associated proteins (DAPs).

The DAPs were subjected to local sequence alignment using NCBI BLAST+ (version 2.2.28+) and InterProScan (version 5.25-64.0) to identify homologous sequences. Subsequent GO term mapping and functional annotation of protein sequences were performed with Blast2GO software (version 4.1.9). After annotation, the proteins were searched against the online KEGG database (https://www.genome.jp/kegg/, accessed on 26 April 2020) to acquire KO numbers and mapped to KEGG pathways. Fisher’s exact test was used for enrichment analysis by comparing GO and KEGG profiles between target and background protein groups. Multiple testing correction was performed via the Benjamini–Hochberg method.

Gene set enrichment analysis (GSEA) was implemented via the clusterProfiler package (v4.18.4) in R (v4.5.2). For each protein with at least one valid LFQ intensity in each group, the FC was then calculated as the ratio of the mean intensity in BTV-oISG15 to that in the NC-oISG15 and log_2_-transformed for GSEA ranking. Missing values were not imputed. Proteins were ranked by their log_2_FC values in descending order for subsequent GSEA. The ovine gene annotation database org.Ovis_aries.eg.db (AH101101) was used as the reference annotation library. Statistical significance was determined using 1000 gene set permutations, and raw *p*-values were adjusted via the Benjamini-Hochberg procedure.

The representative proteins were selected and grouped according to their annotated functional categories and displayed as a functionally grouped heatmap. LFQ intensities were standardized by row-wise z-score across biological replicates. For visualization, sample columns were ordered according to experimental conditions, and no distance-based hierarchical clustering was applied. Selected candidates were subsequently evaluated by endogenous IP-WB.

### 2.7. Western Blot Analysis

To validate the differential enrichment patterns identified by LC–MS/MS, endogenous oISG15 was immunoprecipitated from mock-infected and BTV-infected MDOK cell lysates as described above. The anti-VDAC1 (Abmart, Shanghai, China, Cat. No. T55416F), anti-PDHX (Abmart, Shanghai, China, Cat. No. JC038279F), and anti-SQSTM1/p62 (Abmart, Shanghai, China, Cat. No. F55546) antibodies were used to detect the abundance in the whole-cell lysate input and immunoprecipitated complex.

The signal of each target protein was first normalized to the amount of immunoprecipitated oISG15, and the corresponding input abundance was normalized to β-actin. The relative enrichment index was then calculated as (IP-target/IP-oISG15)/(Input-target/Input-β-actin). The value of the mock-infected control was set to 1 for each target protein.

To examine whether oISG15 was associated with changes in autophagy-related and mitochondrial quality-control markers during BTV infection, MDOK cells were seeded onto a 12-well plate with empty vector or oISG15-HA transfection (1 μg). After 12 h, cells were subjected to BTV-1 infection and chloroquine (CQ, 50 μM) treatment for 24 h to inhibit lysosomel degradation before the sample collection.

The whole-cell lysates were subjected to Western blot analysis for LC3, SQSTM1/p62, global K63-Ub, PINK1, Parkin, BTV NS1, HA-tag, and β-actin. The band intensities of these proteins were normalized to β-actin. For K63-Ub, the integrated signal over the same molecular-weight range was normalized to β-actin. To compare the effect of oISG15 under matched experimental conditions, normalized signals in oISG15-expressing cells were compared with those from corresponding vector control cells. The matched comparisons were oISG15 + CQ versus Vector + CQ, oISG15 + BTV versus Vector + BTV, and oISG15 + BTV + CQ versus Vector + BTV + CQ. The primary antibodies used in this measurement included anti-LC3-II (Abmart, Shanghai, China, Cat. No. T55992F), anti-p62 (Abmart, Shanghai, China, Cat. No. T55546F), anti-K63-Ub (Abmart, Shanghai, China, Cat. No. T56579F), anti-Parkin (Abmart, Shanghai, China, Cat. No. T56641), and anti-PINK1 antibodies (Abmart, Shanghai, China, Cat. No. pk05715). The anti-NS1 antibody was preserved in our laboratory.

## 3. Results

### 3.1. Functional Validation of the Anti-ISG15 Antibody

**Figure 1:**
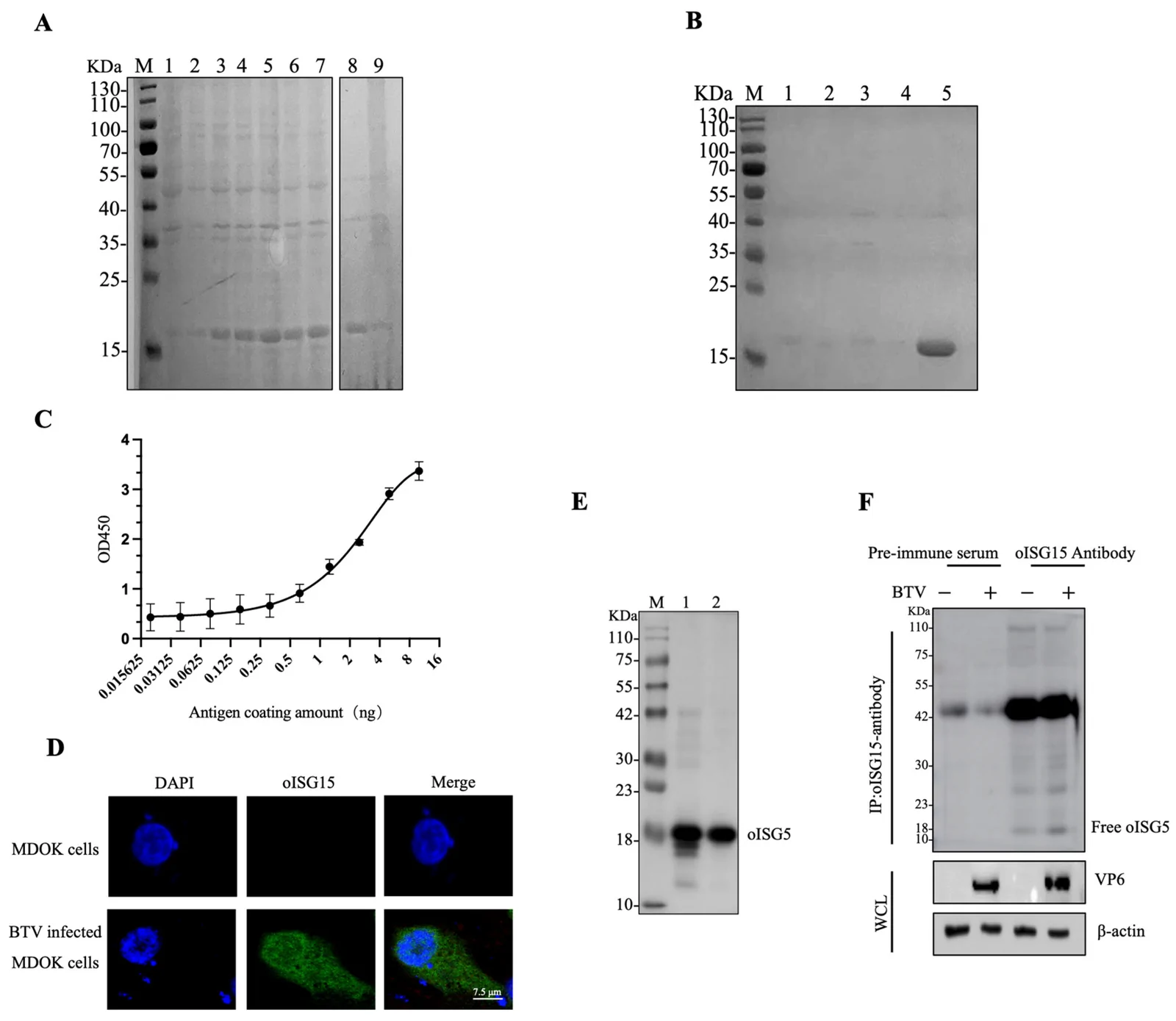
Generation and functional validation of the anti-oISG15 antibody. (**A**). SDS-PAGE analysis for time-gradient induction and solubility of recombinant oISG15 protein. Lane M, pre-stained protein molecular weight marker; Lane 1, bacterial pellets before IPTG induction; Lane 2–7, bacterial pellets harvested after 1–6 h IPTG induction; Lane 8–9, soluble supernatant and insoluble precipitate of bacterial lysate. (**B**). SDS-PAGE detection of Ni-NTA affinity chromatography-purified oISG15. Lane 1–4, wash flow-through fractions; Lane 5, eluted recombinant oISG15 protein. (**C**). Indirect ELISA evaluation of anti-oISG15 antibody reactivity at graded antigen-coating amounts. The purified oISG15 was serially diluted (0.015625–16 ng per well) and coated as a capture antigen, followed by incubation with the anti-oISG15 antibody at a 1:1000 dilution to measure OD_450_ absorbance values of the generated anti-oISG15 antibody. Data are presented as mean ± standard error of the mean (SEM). (**D**). IFA detection for endogenous oISG15 expression in MDOK cells with or without BTV infection. Nuclei were counterstained with DAPI (blue), and oISG15 was visualized with anti-oISG15 antibody (green). The merged images show the cellular localization of oISG15. (**E**). Western blot validation of the generated antibody against recombinant oISG15. Lane 1, total bacterial lysate after 6 h induction; Lane 2, purified recombinant oISG15; the primary oISG15 antibody was used at a 1:10,000 dilution. (**F**). Immunoprecipitation validation of the anti-oISG15 antibody. MDOK cells were mock-infected or infected with BTV-1, and cell lysates were immunoprecipitated with anti-oISG15 antibody or pre-immune rabbit serum as a negative control. The input lysates and immunoprecipitates were analyzed by Western blot using anti-oISG15 antibody; primary antibody:anti-oISG15 antibody (1:5000 dilution).

As shown in Figure 1A, distinct protein bands consistent with 18 kDa gradually emerged in bacterial samples collected at 1–6 h post-induction (lane 2–7). After bacterial lysis, the dominant form of the induced oISG15 protein was detected in the supernatant (lane 8), but not in the precipitation fraction (lane 9). Following loading of the oISG15-containing supernatant onto the Ni-NTA affinity column, the resin was washed four times with wash buffer (lane 1–4), and the bound recombinant oISG15 was subsequently eluted (lane 5) (Figure 1B).

The antigen reactivity of the generated anti-oISG15 antibody was verified by indirect ELISA, IFA, Western blot, and immunoprecipitation. The OD_450_ values increased with increasing antigen-coating amounts, confirming dose-dependent recognition of recombinant oISG15 by the generated antibody (Figure 1C). The IFA assay showed that endogenous oISG15 was detected in BTV-infected MDOK cells and was distributed in both the cytoplasm and nucleus, whereas little or no signal was observed in uninfected MDOK cells (Figure 1D). The recombinant oISG15 from induced bacterial lysates and the purified oISG15 protein were readily detected with the anti-oISG15 antibody via Western blot, confirming that this antibody could recognize the linear epitopes of oISG15 (Figure 1E).

Subsequently, we examined whether the anti-oISG15 antibody could efficiently immunoprecipitate endogenous oISG15. As shown in Figure 1F, the anti-oISG15 IP produced a clear oISG15 signal from MDOK cell lysates. In contrast, little or no oISG15 signal was recovered using pre-immune serum under the corresponding conditions. These results indicated that this antibody could immunoprecipitate endogenous oISG15 under native conditions and was suitable for subsequent enrichment of oISG15-associated protein complexes.

### 3.2. Identification and Functional Enrichment of Differential oISG15-Associated Proteins During BTV Infection

**Figure 2:**
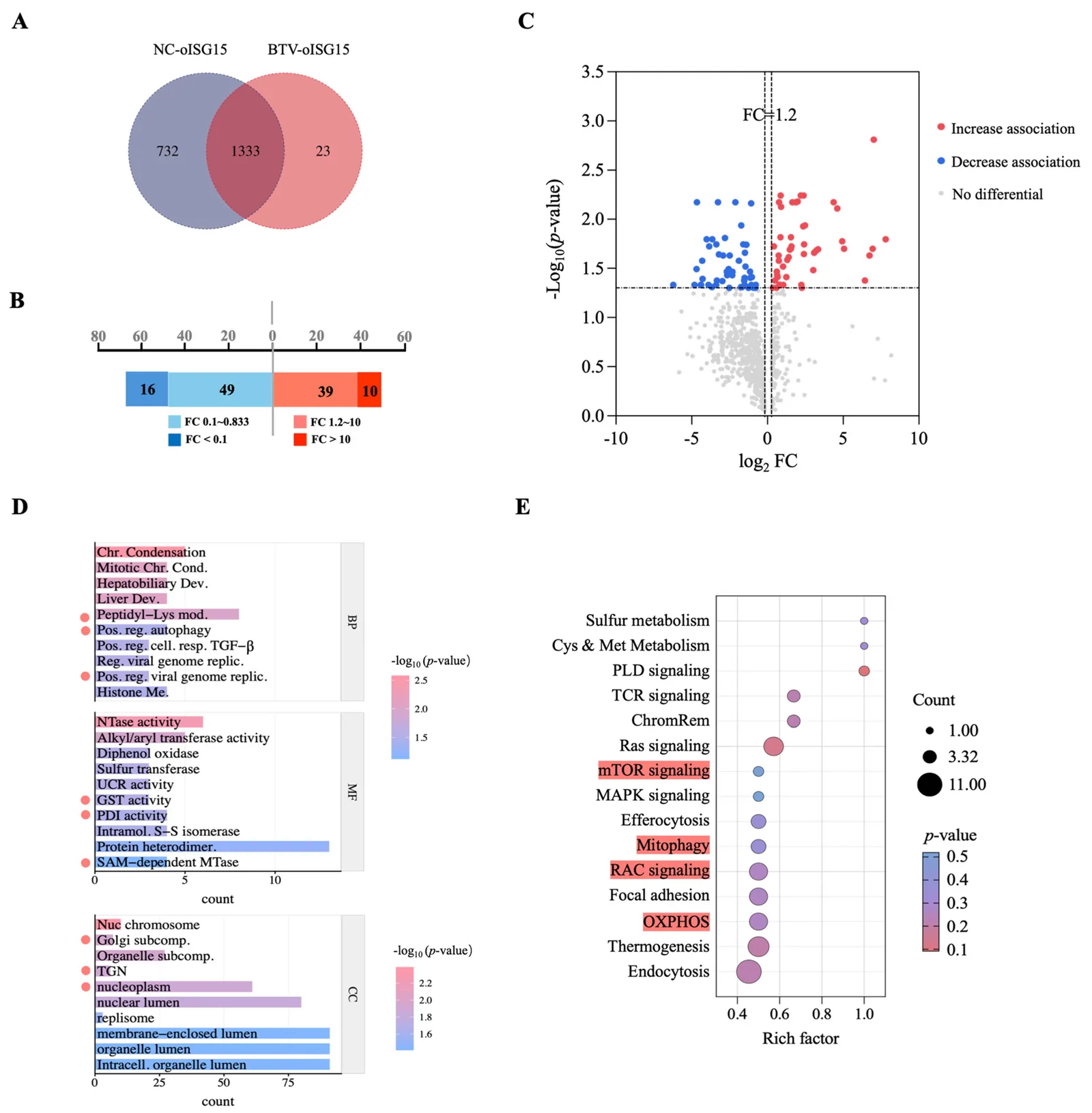
Identification and Functional Enrichment Analysis of Differential Associated Proteins upon BTV Infection. (**A**). The Venn diagram represents proteins detected in at least one replicate, whereas qualitative group-specific proteins used for downstream analyses were defined according to the ≥2 out of 3 replicate criterion described in the Section Materials and Methods. (**B**). Numbers of significantly differentially enriched proteins showing higher or lower association with oISG15 in the BTV-oISG15 group relative to the NC-oISG15 group. Proteins were grouped according to the indicated FC ranges. Statistical testing was performed using a two-sided Student’s *t*-test on LFQ intensity values, followed by Benjamini-Hochberg correction for multiple testing. Proteins with FDR < 0.05 and FC ≥ 1.2 or ≤0.833 were considered significantly differentially enriched. (**C**). Volcano plot of quantitatively detected proteins. The volcano plot displays raw *p*-values. The pink and blue dots were significantly differentially enriched proteins, which were mentioned in (**B**). (**D**). GO enrichment analysis of DAPs. The enrichment plot was visualized according to raw *p*-values. (n = 662). (**E**). Global dot plot of KEGG enrichment among the DAPs (n = 662). The color scale indicated different thresholds of the raw *p*-value, and the size of the dot indicated the number of genes corresponding to each pathway.

IP coupled with LC–MS/MS was used to characterize proteins associated with oISG15 during the BTV infection. A total of 1333 proteins were detected in both groups. A further 732 and 23 proteins were detected exclusively in the NC-oISG15 and BTV-oISG15 groups, respectively, based on detection in at least one biological replicate (Figure 2A). These proteins were further filtered according to the predefined criterion of presence in ≥2 out of 3 replicates in one group and absence in the other for subsequent classification as qualitative group-specific proteins. Among proteins quantitatively detected in both groups, 65 showed significantly decreased enrichment in BTV-oISG15 immunoprecipitates, including 16 with strong decreases (FC < 0.1) and 49 with moderate decreases (FC = 0.1–0.833). In contrast, 49 proteins showed increased enrichment, including 39 with strong increases (FC > 10) and 10 with moderate increases (FC = 1.2–10) (Figure 2B). The volcano plot further illustrated the overall distribution of these quantitative changes (Figure 2C).

The GO enrichment analysis showed that the DAPs were enriched in positive regulation of autophagy, peptidyl-lysine modification, and positive regulation of viral genome replication; molecular functions such as glutathione S-transferase activity (GST activity), protein disulfide isomerase activity (PDI activity), and S-adenosylmethionine-dependent methyltransferase activity (SAM-dependent methyltransferase); the cellular components mainly include nucleoplasm, Golgi subcompartment, and trans-Golgi network (TGN) (Figure 2D). The KEGG analysis revealed the enrichment in pathways including oxidative phosphorylation, mitophagy-animal, and mTOR signaling, as well as pathways related to endocytosis, MAPK signaling, and regulation of actin cytoskeleton (Figure 2E).

### 3.3. Functional Redistribution of the oISG15 Association Landscape During BTV Infection

**Figure 3:**
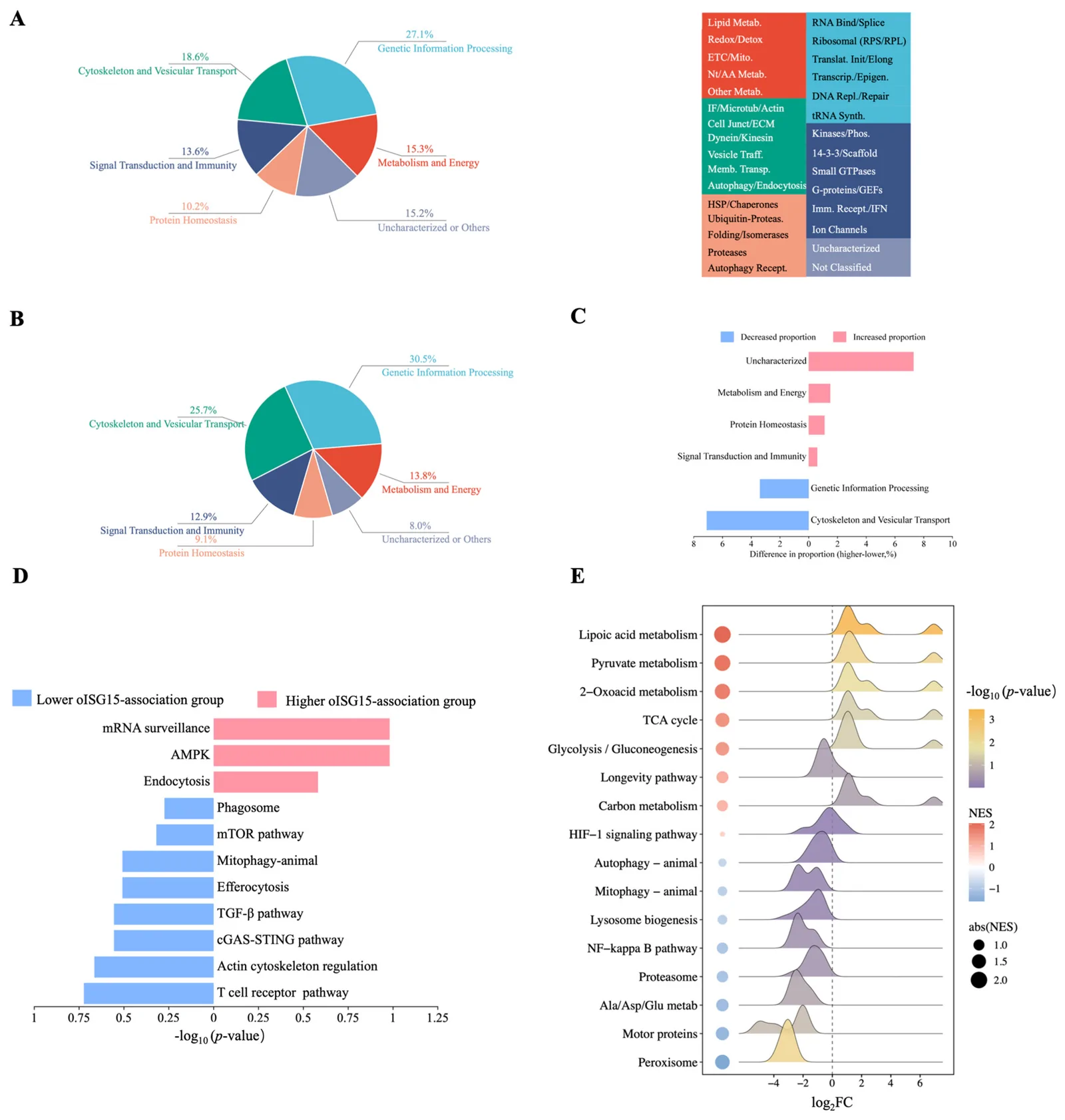
BTV remodeled oISG15 association with cytoskeletal, autophagic vesicle components, and metabolic regulators. (**A**,**B**). Functional distribution of the higher (n = 59) and lower (n = 603) oISG15-association groups. Proteins were categorized into six major functional classes. Data were presented as the percentage of each category within the groups, and the subcategories of each main functional class were listed and stained with corresponding colors. (**C**). Differential analysis of functional class between the higher and lower association. The vertical axis represented the difference in proportion, calculated as (higher% − lower%). Positive values (pink) indicate functional classes relatively enriched in the higher-association group, with negative values (blue) for the lower-association group. (**D**). Pathway enrichment of the higher- and lower-enrichment groups, plotted as −log_10_ (*p*-value). (**E**). GSEA ridge plot showed the enrichment of all quantified proteins (present in ≥ 1 out of 3 replicates in both BTV-oISG15 and NC-oISG15 groups, n = 1333) pathways. The log_2_FC was displayed by ridge distribution on the horizontal axis. The vertical axis showed the names of enriched pathways and their normalized enrichment score (NES). Color intensity of the ridge represented the −log_10_ (*p*-value). The gene sets were derived from the KEGG database.

To characterize the functional patterns associated with BTV infection, the DAPs were divided into higher- and lower-enrichment groups according to their relative enrichment in BTV-oISG15 immunoprecipitates. In the higher group, genetic information processing constituted the largest proportion (27.1%), followed by cytoskeleton and vesicular transport (18.6%), metabolism and energy (15.3%), signal transduction and immune-related proteins (13.6%), and protein homeostasis (10.2%) (Figure 3A). Within the lower group, genetic information processing remained the most enriched functional class (30.5%). Notably, cytoskeleton and vesicular transport accounted for the particular higher fraction (25.7%) relative to that observed in the higher group (Figure 3B). The differential proportional analysis of these two groups (Δ% = percentages in higher oISG15-association group−percentages in lower oISG15-association group) further uncovered a functional bias in oISG15 association remodeling. The cytoskeleton and vesicular transport category displayed the most pronounced negative shift (Δ= −7.1%), demonstrating that structural proteins and autophagic vesicle-associated components showed lower enrichment from the oISG15 association following BTV infection. By contrast, the uncharacterized category exhibited the greatest positive enrichment (Δ = +7.3%), indicating that BTV-induced oISG15 association remodeling extends beyond characterized pathways. These poorly annotated proteins may warrant further investigation during BTV infection (Figure 3C).

Proteins showing higher association with oISG15 tended to be involved in mRNA surveillance, AMPK, and endocytosis pathways, while proteins with lower association tended to be involved in T cell receptor signaling, actin cytoskeleton regulation, TGF-β, and other pathways (Figure 3D). To independently evaluate pathway-level remodeling changes across the quantified proteome, we performed GSEA on all quantified proteins ranked by their LFQ FC (BTV-oISG15/NC-oISG15). Eight gene sets governing mitochondrial energy metabolism were markedly enriched in the positive direction, encompassing lipoic acid metabolism, pyruvate metabolism, 2-oxocarboxylic acid metabolism, the TCA cycle, and other pathways. Conversely, eight functional modules linked to autophagic turnover and innate immune responses exhibited negative enrichment, including autophagy, mitophagy, lysosome biogenesis, NF-κB, motor protein function, and others (Figure 3E).

Together, the KEGG and GSEA results indicated differential functional enrichment within the oISG15-associated proteome, characterized by lower enrichment of autophagy/lysosome-related proteins and higher enrichment of mitochondrial metabolism-related proteins in BTV-oISG15 immunoprecipitates.

### 3.4. BTV Remodels oISG15 Associations and oISG15 Alters Autophagy-Related Markers During BTV Infection

**Figure 4:**
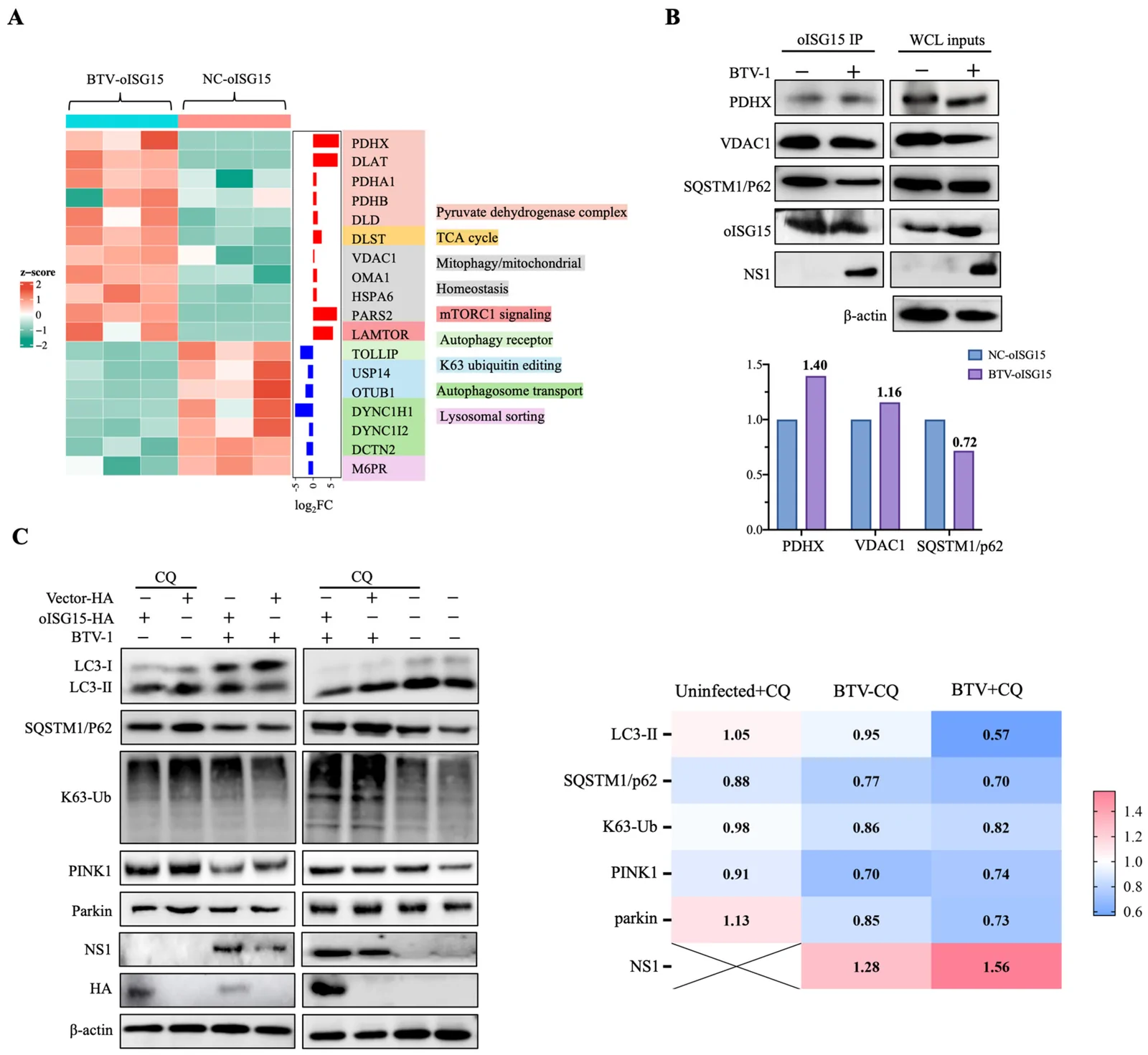
BTV remodels the oISG15 association and modulates autophagy-related signaling. (**A**). Functionally grouped heatmap depicting the relative abundances of selected oISG15-associated proteins in BTV-oISG15 and NC-oISG15 group. Proteins from pathway enrichment analyses were screened by functional relevance and grouped into predefined functional categories. LFQ intensities were standardized by row-wise z-score across biological replicates. (**B**). Targeted examination of PDHX, VDAC1, and SQSTM1/p62 co-enrichment with oISG15 from independent biological experiments. The endogenous oISG15 was immunoprecipitated from BTV-infected and mock-infected MDOK cells. Quantification represents the relative enrichment index calculated as (IP-target/IP-oISG15)/(Input-target/Input-β-actin), and the value of the mock-infected control was set to 1 for each protein. (**C**). The MDOK cells were subjected to the indicated combinations of vector or oISG15-HA transfection, BTV infection, and CQ treatment. The whole-cell lysates were immunoblotted for LC3, SQSTM1/p62, global K63-Ub, PINK1, Parkin, NS1, HA-tag, and β-actin. Values of heatmap show the oISG15/vector ratios under matched experimental conditions after normalized by β-actin. The immunoblots of (**B**) and (**C**) show the representative of independent biological replicates, and the densitometric values presented correspond to these representative experiments. Similar trends were observed in repeated experiments.

To pinpoint individual proteins underlying pathway reprogramming, the selected representative molecules related to mitochondrial metabolism, autophagy/lysosome function, intracellular transport, and immune signaling were displayed in a functionally grouped heatmap. Proteins showing higher enrichment in BTV-oISG15 immunoprecipitates mainly included mitochondrial metabolic proteins: all five PDC subunits (PDHX, DLAT, PDHA1, PDHB, DLD), TCA cycle enzyme DLST, mitochondrial outer-membrane protein VDAC1, mitochondrial protease OMA1, and mTORC1-lysosome scaffold LAMTOR4. In contrast, proteins showing lower enrichment included autophagy receptors (TOLLIP), K63-specific deubiquitinases (USP14 and OTUB1), dynein dynactin complex components (DYNC1H1, DYNC1I2, DCTN2), and lysosomal sorting receptor M6PR. SQSTM1/p62, SNAP29, and LAMTOR1 also showed lower enrichment in BTV-oISG15 immunoprecipitates (Figure 4A). Based on the association profiles and functional relevance, PDHX, VDAC1, and SQSTM1/p62 were prioritized for subsequent validation. PDHX and VDAC1 showed higher relative co-enrichment with oISG15, whereas SQSTM1/p62 showed lower relative co-enrichment, consistent with the IP–MS trends (Figure 4B). The endogenous IP assay indicated that BTV infection enhanced the relative enrichment of oISG15 with PDHX and VDAC1, whereas it reduced its association with SQSTM1/p62.

To further determine whether the effect of oISG15 on autophagy-associated signaling was constitutive or preferentially manifested during BTV infection, the analyses matching vector control and oISG15-overexpression conditions were performed in the presence or absence of BTV and CQ. The levels of PINK1 and Parkin were examined as mitochondrial quality-control markers, and BTV NS1 was included as a viral protein readout. The experimental design was as follows: uninfected + CQ (oISG15/vector + CQ), BTV-CQ (oISG15/vector + BTV), and BTV + CQ (oISG15/vector + BTV + CQ).

In the uninfected cells treated with CQ, oISG15 overexpression had only modest effects on the examined markers. Thus, oISG15 alone had relatively little effect on these markers under lysosomal inhibition. During BTV infection in the absence of CQ, several markers showed lower oISG15/vector ratios, with the larger decreases observed for SQSTM1/p62 and PINK1, whereas LC3-II changed only modestly. This pattern suggested that the effects associated with oISG15 overexpression were more evident during BTV infection. A similar and generally more pronounced pattern was observed under CQ treatment. LC3-II and SQSTM1/p62 exhibited greater reductions relative to their matched vector controls, while global K63-Ub decreased to a lesser extent (Figure 4C).

The accompanying decreases of PINK1 and Parkin suggested that oISG15 overexpression was associated with altered mitochondrial quality-control markers during BTV infection. However, these changes do not establish altered mitophagic flux. NS1 abundance was increased in oISG15-overexpressing cells relative to vector controls in both the absence and presence of CQ.

## 4. Discussion

In this study, we generated an anti-oISG15 antibody suitable for endogenous IP to characterize the oISG15-associated proteome. We found that BTV infection was accompanied by lower oISG15 association with several autophagy- and trafficking-related proteins and enhanced association with mitochondrial metabolic proteins. Functional analyses further showed that oISG15 overexpression exerted limited effects on autophagy-associated markers in uninfected cells. Upon BTV infection, however, oISG15 overexpression attenuated LC3-II, SQSTM1/p62 and global K63-Ub signals. Meanwhile, the abundance of PINK1/Parkin altered with a consistent trend, accompanied by increased abundance of viral NS1 protein. Collectively, these findings identified a BTV-dependent remodeling of the oISG15 association landscape that coincides with an altered autophagy-related phenotype.

ISG15 exists at low levels under normal physiological conditions, yet its transcription is significantly upregulated when interferon regulatory factors (IRFs) associate with promoters harboring interferon-stimulated response elements (ISREs) [14]. In a study of *Chlamydia*-induced ISG15 expression, basal ISG15 expression was detected by Western blot in uninfected and IFN-untreated epithelial cells [15]. IP–MS-based studies have largely focused on target proteins and their cellular interaction partners. Mao et al. employed antibodies targeting the SARS-CoV-2 nucleocapsid protein to screen interacting proteins via IP–MS, and anti-NP1 rabbit antibodies were used to identify host cell proteins during the minute virus of canines (MVC) infection [16,17]. Antibodies specific to human and mouse ISG15 were exploited to characterize the ISG15-conjugated substrates in human monocytes and USP18-deficient mouse embryonic fibroblasts [18]. Liu et al. developed a monoclonal swine ISG15 antibody to measure the function of extracellular ISG15; subsequently, they adopted it to profile the ISGylation candidates [19,20].

Here, using the oISG15 antibody to perform the IP combined with LC–MS/MS analysis, we found that a broad range of proteins was co-enriched with oISG15. The antibody in our study cannot selectively recognize the ISG15-substrate conjugation. In contrast to ubiquitination, whose linkage types are well characterized and enable the generation of linkage-specific antibodies capable of distinguishing K48- and K63-Ub conjugates [10,21]. Accordingly, the proteins identified here are referred to primarily as oISG15-associated proteins rather than direct oISG15-binding partners. In addition, protein abundance measured by IP–MS reflects the relative enrichment level and may also be influenced by differences in bait recovery between conditions. Therefore, the representative candidates were further evaluated by IP verification after normalization to immunoprecipitated oISG15 and the corresponding input abundance. The results supported changes in association with PDHX, VDAC1, and SQSTM1/p62, but likewise did not by themselves establish direct molecular binding.

The large number of oISG15-associated proteins is not a special case. Gan, J’s research showed that 2829 proteins were identified in IFN-β-treated HAP1 cells, followed by the IP with human ISG15 antibody and label-free quantitative proteomics analysis [22]. These proteins were strongly enriched for enzymes functioning in metabolic pathways and located in the cytoplasm and mitochondria, which was consistent with our finding that oISG15 redistributed its association toward metabolic pathways. Zhang et al. found that basal and infection-induced autophagy were increased in the enhanced ISGylation model compared to the ISG15 deficiency model by quantitative label-free proteomics [23]. However, in our study, the oISG15 association with autophagy-related was diminished during BTV infection. This apparent discrepancy, where enhanced ISGylation promotes autophagy yet oISG15 overexpression was associated with an attenuated BTV-related autophagic phenotype, prompted us to consider whether oISG15 operates through an ISGylation-independent mechanism.

The present association profile provided a potential connection between the previously reported effects of oISG15 on viral-protein stability and the autophagy-related phenotype. BTV infection was associated with lower oISG15 association with autophagy receptors and trafficking components, including TOLLIP, SQSTM1, K63-specific deubiquitinases (USP14, OTUB1), and the dynein-dynactin transport complex. Such redistribution could alter the accessibility or organization of ubiquitinated cargo within selective-autophagy pathways [24]. However, the present data do not identify the relevant K63-ubiquitinated substrates or demonstrate that altered oISG15 association directly prevents their autophagic recognition. The reduction in global K63-Ub observed following oISG15 overexpression should therefore be interpreted as a pathway-level change rather than evidence for modification of specific viral or mitochondrial substrates. USP14 is known to negatively regulate autophagy initiation by removing the K63 ubiquitin chain on Beclin 1, while OTUB1 can stabilize its interaction with mTOR by deubiquitinating DEPTOR during nutrient deficiency, thus allowing autophagy induction [25,26]. Although the LC3-II and SQSTM1/p62 responses observed in the presence and absence of CQ support an alteration in autophagy-related processes, these measurements do not fully resolve autophagic flux affected by oISG15. Tandem fluorescent LC3 reporters, ultrastructural analysis, and complementary lysosomal inhibition approaches will be required to address this question directly. The specific process and mechanisms remain to be further verified.

The dynein-dynactin transport complex is the main motor complex that drives autophagosome transport along microtubules to the center of cells [27]. The PDC is the “gatekeeper” connecting glycolysis and the TCA cycle. It is the main entrance of carbon into aerobic metabolism. VDAC1 is the most abundant pore protein on the outer membrane of mitochondria. It controls the entry and exit of metabolites, ions, and Ca^2+^ into mitochondria [28,29]. Another prominent feature of the proteomic changes was the increased enrichment of mitochondrial metabolic proteins in oISG15 immunoprecipitates, including all five PDC subunits and VDAC1, suggesting that oISG15 is associated with protein complexes linked to mitochondrial carbon metabolism. However, mitochondrial respiration, membrane potential, PDC activity, and metabolic flux were not measured in the present study. Therefore, whether the altered oISG15 association profile produces functional changes in mitochondrial metabolism remains unresolved. The accompanying changes in PINK1 and Parkin in our study indicate that oISG15 was associated with altered mitochondrial quality-control markers during BTV infection. However, total cellular PINK1 and Parkin abundance do not represent their mitochondrial recruitment, activation state, or mitophagic flux.

The recent live-cell imaging study indicated that NS1 tubules form aggresomes at the microtubule-organizing center [30]. Aggresomes are known to selectively remove the misfolded proteins into the aggresome–autophage pathway, and numerous viral proteins have been shown to be cleared by this route [31]. However, BTV infection has been known to trigger autophagic flux through the Akt/AMPK/mTOR axis, thereby supplying membrane scaffolds and metabolic intermediates for viral replication [32]. In the present study, oISG15 overexpression was accompanied by both attenuation of BTV-associated LC3-II/SQSTM1-p62 accumulation and increased NS1 abundance. These findings reconnect the autophagy-associated phenotype with the previously reported proviral activity of oISG15. However, because autophagy was not independently manipulated to determine whether the NS1 increase depends on this pathway, the present data establish an association rather than a causal link between altered autophagy and enhanced viral protein abundance.

Collectively, our data showed that BTV infection was accompanied by a redistribution of the oISG15-associated proteome: lower enrichment of autophagy/trafficking-related components and higher enrichment of mitochondrial metabolic proteins. This remodeling occurs in parallel with an oISG15-associated attenuation of BTV-associated autophagy-related markers, changes in mitochondrial quality-control markers, and increased NS1 abundance. At present, these observations establish coordinated associations rather than a causal pathway connecting mitochondrial association, autophagy regulation, and viral replication. Targeted perturbation of PDHX, VDAC1, SQSTM1/p62, TOLLIP, or other candidate proteins, together with measurements of viral replication, will be required to determine whether these proteins functionally mediate the oISG15-dependent phenotype during BTV infection. Finally, because the present study was performed in MDOK cells, further validation in primary ovine cells is needed to assess the broader physiological relevance of these findings.

## 5. Conclusions

This study revealed that BTV infection remodels the oISG15-associated proteome, characterized by lower association with several autophagy- and trafficking-related proteins and higher association with mitochondrial metabolic proteins. The endogenous IP supported the higher association of oISG15 with PDHX and VDAC1 and lower association with SQSTM1/p62. Functionally, oISG15 overexpression was associated with attenuated BTV-induced changes in LC3-II, SQSTM1/p62, and global K63-Ub, together with alterations in PINK1/Parkin abundance and increased viral NS1. However, the causal relationship among these processes remains to be determined. The present study therefore provides a framework for future mechanistic investigation of how oISG15-associated host proteins contribute to BTV infection.

## Data Availability

The mass spectrometry proteomics data have been deposited to the ProteomeXchange Consortium (https://proteomecentral.proteomexchange.org) via the iProX partner repository [33,34] with the dataset identifier PXD082827.
